# Comparison of Accelerated and Standard Hepatitis B Vaccination Schedules in High-Risk Healthy Adults: A Meta-Analysis of Randomized Controlled Trials

**DOI:** 10.1371/journal.pone.0133464

**Published:** 2015-07-21

**Authors:** Hui Jin, Zhaoying Tan, Xuefeng Zhang, Bei Wang, Yueyuan Zhao, Pei Liu

**Affiliations:** 1 Department of Epidemiology and Health Statistics, Southeast University, Nanjing, China; 2 Key Laboratory of Environmental Medicine Engineering, Ministry of Education, School of Public Health, Southeast University, Nanjing, China; 3 Jiangsu Provincial Centre for Disease Control and Prevention, Nanjing, China; The University of Tokyo, JAPAN

## Abstract

**Background:**

Selecting the most efficient vaccination schedule is an important issue.

**Objective:**

To assess the beneficial and harmful effects of accelerated hepatitis B vaccination schedules in high-risk healthy adults.

**Methods:**

We searched controlled trial registers of The Cochrane Library as well as MEDLINE, EMBASE, VIP Database for Chinese Technical Periodicals, and the Chinese National Knowledge Infrastructure databases for randomized controlled trials published up to December 2013 that compared accelerated hepatitis B vaccine schedules to the standard schedule in adults. The results were presented as relative risk with 95% confidence intervals. Fixed or random effect models were used for analysis.

**Results:**

We identified 10 randomized trials, all with one or more methodological weaknesses. Compared to the standard schedule, most accelerated schedules resulted in higher proportions of healthy vaccines more rapidly reaching anti-hepatitis B antibody levels >10 IU/L (*P*<0.05) initially and maintaining similar seroprotection rates after 6 months (*P*>0.05). Although accelerated schedules produced anti-hepatitis B levels higher than the standard schedule for the first month after the initial vaccine dose, they were significantly lower than the standard schedule after 6 months, except for an accelerated schedule that called for a fourth booster injection 12 months after the initial dose. Subjects administered accelerated vaccine schedules had similar compliance rate as those administered the standard schedule over the first 6 months of vaccination (relative risk = 1.00, 95% confidence interval: 0.84–1.21).

**Conclusion:**

For rapid seroconversion and almost immediate short-term protection, accelerated vaccination schedules could be useful for at-risk groups. However, additional studies on the long-term protection and effectiveness of the primary doses of accelerated schedules are necessary.

## Introduction

Hepatitis B is a globally distributed acute and chronic communicable disease associated with cancers and major hepatic diseases. Chronic hepatitis B virus (HBV) infections can lead to hepatocellular carcinoma (HCC), cirrhosis, and death [1]. Although hepatitis B vaccination offers high safety, cost-effectiveness and has resulted in a significant worldwide decline in hepatitis B incidence in children and adolescents, the vaccine has not been sufficiently utilized in adults, especially high-risk groups, leaving them susceptible to HBV-related complications [2, 3].

Worldwide, the standard hepatitis B vaccination schedule for adults consists of three doses administered on a 0–1–6 month schedule, which typically results in at least 85% seroprotection in target groups [4]. Unfortunately, despite longstanding recommendations, it remains difficult to reach at-risk groups due to some factors including lack of self-protection cognition and limited healthcare programs targeting certain high-risk groups such as injection drug users and prisoners. Furthermore, even when they can be reached, those who engage in high-risk behaviors often fail to comply with the required hepatitis B vaccination regimen.

The need for accelerated hepatitis B vaccination schedules for specific at-risk groups is well recognized. An accelerated vaccination schedule in a small group of healthy individuals has been shown to rapidly induce protective antibody titers [5], and accelerated post-exposure prophylaxis alone without hepatitis B immune globulin (HBIG) has been suggested to offer equally effective protection [6]. However, one major concern with accelerated vaccination schedules is whether the protection persists similarly to standard vaccination schedules. Moreover, various short schedules, such as 0–1–2 months [7–14], 0–1–2–6 months [15, 16], 0–1–2–12 months [15, 17–22], 0–1–12 months [20, 23], 0–1–4 months [24, 25], 0–14–42 days [26], 0–7–21 days [27–31], 0–7–28–56 days [4], and 0–7–21–360 days [32], administered to medical students, health-care workers, prisoners, drug users, dialysis patients, and patients with HIV complicate determination of the optimal choice of an accelerated schedule.

Several schedules [24, 32, 33] have been investigated to enhance compliance or more quickly induce protective antibody levels without reducing the hepatitis B vaccination immunogenicity. Herck et al. [32] found that accelerated (0–1–2–12 months) or super-accelerated schedules (0–7–21–360 days) resulted in higher proportions of vaccines reaching anti-hepatitis B antibody (anti-HM) levels >10 IU/L more rapidly. A fourth dose at month 12 is still required due to lack of the long-term protection data of these accelerated schedules. However, completing the schedule with a fourth dose is more difficult to ensure the compliance of hard-to-reach populations than completing a standard 0–1–6 month schedule.

When hepatitis B immunization programs targeting at-risk groups are implemented or evaluated, selecting the most efficient vaccination schedule is an important issue. It is beyond doubt that any adaptations should aim to optimize immunization program compliance while maintaining the vaccine’s immunogenicity and efficacy. Since people in these at-risk populations most often continue to be at risk, long-term protection against hepatitis B is important. This paper reviews available RCT evidence on accelerated hepatitis B vaccination schedules vs. the standard schedule for high-risk groups to assess beneficial and harmful effects.

## Material and Methods

### Search strategy

We searched MEDLINE, EMBASE, The Cochrane Library, Chinese National Knowledge Infrastructure (CNKI), and the VIP Database for Chinese Technical Periodicals databases without language restrictions for relevant randomized controlled trials (RCTs) published between January 1980 and December 2013. The search terms included (“vaccine” or “vaccination”) and (“hepatitis B” or “HBV” or “hepatitis B virus”) and “schedule”. The bibliographies of the original studies, reviews, and relevant conference abstracts were manually searched.

### Inclusion and exclusion criteria

We included studies with randomized controlled trials (RCTs) in their design or epidemiologic methods. High-risk healthy subjects more than 15 years of age without previous hepatitis B infections and negative for serum hepatitis B markers, including hepatitis B surface antigen (HBsAg), anti-HBs, or hepatitis B core antibody (HBcAb), were included. High risk adult meant those who were in contact with blood or blood products, blood contaminated instruments, stained body fluids, or tissues, including medical students, health-care workers, prisoners, drug users, etc. Only comparisons of accelerated schedules (≥3 doses) to the standard schedule (0–1–6 months) were assessed. Antibody levels or protective rates between the groups were compared at the same elapsed time after the initial dose.

We excluded quasi-randomized trials and observational studies. Only the most recent or detailed study was chosen for repeated published studies.

### Data extraction and outcome definitions

Two researchers (HJ and ZT) independently selected relevant studies and made post-hoc assessments of methodological quality using The Cochrane Library study quality evaluation tool [34]. We extracted the following characteristics from each RCT: primary author, publication year, number of randomized subjects, methodological quality, intervention regimens, doses and vaccine types, routes of vaccine injection, vaccination schedules, mean age, proportion of males, duration of follow-up, outcome measures, and number and type of adverse events in both intervention and control groups.

The primary outcome measures were hepatitis B protective events at follow-up. A hepatitis B protective event was defined as two or more consecutive patient blood specimens positive for anti-HB levels above 10 IU/L, a level considered protective against HBV infection, several months after initial vaccine dose or at maximum follow-up. Secondary outcome measures were: (1) Anti-HB antibody levels, either expressed as geometric mean titers (GMT) or mean titers; (2) Compliance rates defined as the proportion of participants in each group who completed the full vaccination course according to each protocol; and (3) Any localized or systemic adverse events.

### Quality assessment

Study quality was evaluated using standards recommended by the Cochrane Handbook for Systematic Reviews of Interventions version 5.1.0, including random sequence generation, allocation concealment, blinding, incomplete outcome data, selective reporting, and other biases. The risk of bias was considered high when high bias existed in any domain, low if all key domains (all domains except random sequence generation and allocation concealment) were low bias, and unclear in all other cases. Two authors, HJ and ZT, assessed bias risks independently; disagreements were resolved with the help of a third author (PL). The Preferred Reporting Items for Systematic Reviews (PRISMA) checklist is shown in S1 Data.

### Statistical analysis

Analyses for binary outcomes included all patients, irrespective of compliance or follow-up (intention-to-treat, ITT). Per-protocol (PP) analysis was also considered for seroprotection rates. Estimated pooled relative risk (RR) and 95% confidence interval (95% CI) were determined using the Mantel–Haenszel fixed effects model. We used a random-effects inverse variance model when we detected substantial statistical heterogeneity. For anti-HB level analysis, we log transformed data for all included studies and performed a meta-analysis on the log-scale mean differences. We tested heterogeneity using the chi-square test and I^2^. I^2^ scores of 25%, 50%, and 75%, indicated low, moderate, and high degrees of heterogeneity, respectively. *P* values <0.10 in the chi-square test indicated heterogeneity between studies.

We planned the following subgroup analyses: (1) Methodological quality. We planned to divide trials into high quality (i.e., trials with low risk of bias) and low quality (i.e., trials with higher risk of bias); (2) Hepatitis B events in relation to follow-up duration; (3) Different types of accelerated schedules. We tested for differences between estimates of intervention effects with best interactions. Funnel plots were used to check for publication bias. For all tests, 95% CIs in RR not including “1” or 95% CIs in mean difference not including “0” indicated statistical significance. We used RevMan 5.0 (Copenhagen: Nordic Cochrane Centre, The Cochrane Collaboration, 2011) for statistical analysis.

## Results

**Fig 1:**
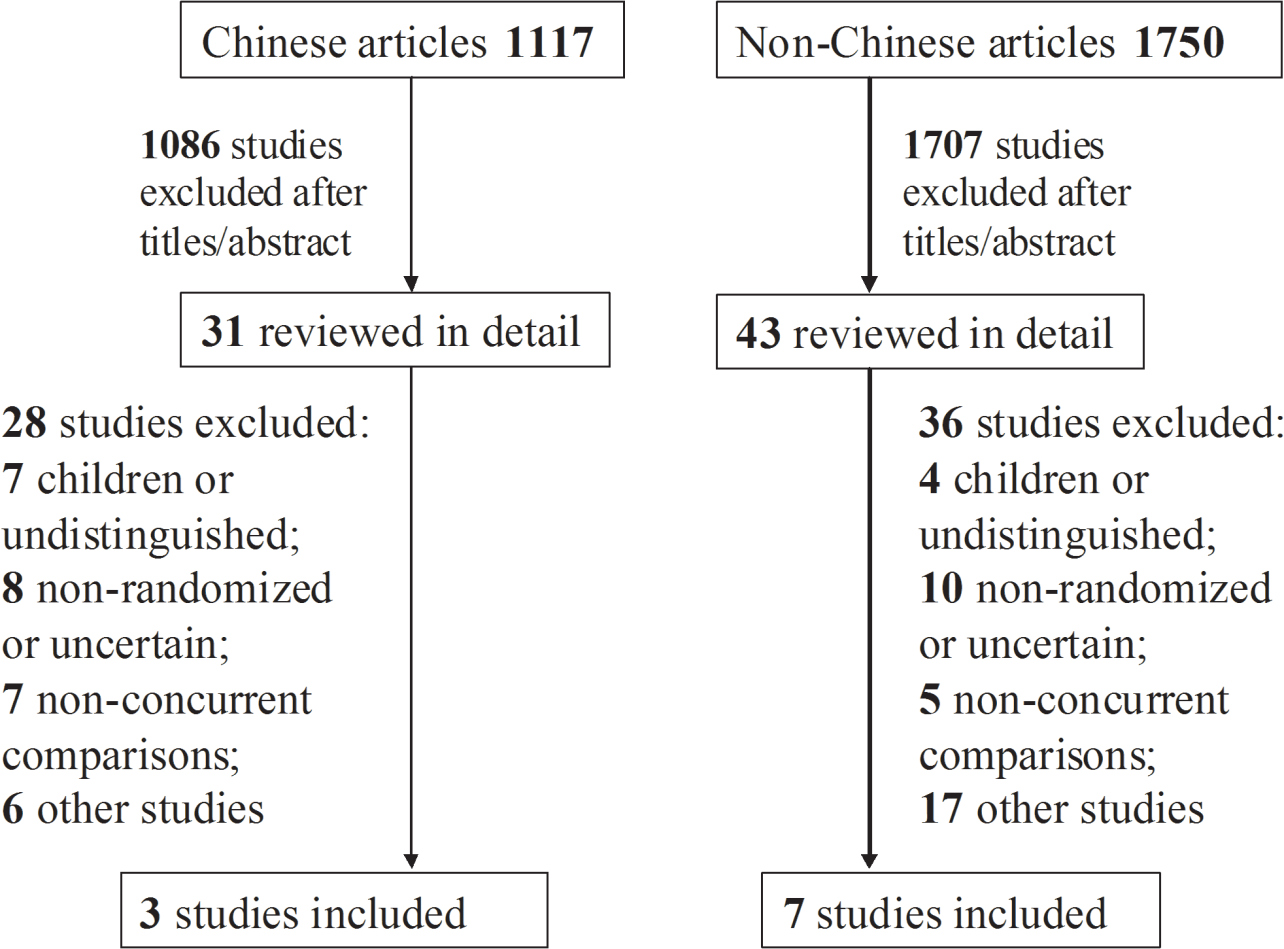
Flow chart of included studies.

A total of 2,867 titles and abstracts were screened and 74 full articles retrieved (Fig 1). The retrieved articles included three trials in Chinese [29–31], six in English [4, 13, 18, 19, 22, 26], and one in Italian [21]. Excluded studies and the reasons for their exclusion are listed in S2 Data. The characteristics of the studies included in our analyses are shown in Tables 1 and 2. Most study subjects were healthy medical students [22, 26, 29] and healthy adults [13, 18, 19, 21, 22, 30, 31], and only one study included male prisoners [4].

**Table 1:** Overview of studies according to vaccination schedule in different at-risk populations.

Ref.	Vaccine	Subjects	Age (male/female)	Schedule	Group	Sample size	Adverse events
Yuan2004[31]	RV	Military men^a^	15–20 y (100/0)	0–7–21 days	T	50	Unclear
	10 ug/dose			0–1–6 months	C	50	
Chen2006[29]	RV	Medical students^a^	15–21 y	0–7–21 days	T	100	Fever and injection
	10 ug/dose		(65/135)	0–1–6 months	C	100	site pain
Yuan2006[30]	RV	Military men^a^	18–50 y (300/0)	0–7–21 days	T	150	Fever and injection
	10 ug/dose			0–1–6 months	C	150	site pain
Wahl1988[26]	RV	Non-pregnant medical students^a^	18–40 y (0/53)	0–14–42 days	T	27	Unclear
	10 ug/dose			0–1–6 months	C	26	
Ricciardi1990[21]	RV	Health care workers	NR (35/80)	0–1–2–12 months	T	50	Unclear
	20 ug/dose			0–1–6 months	C	65	
Hess1992[22]	RV	Medical students	18–73 y	0–1–2–12 months	T	143	Headache, diarrhea
	20 ug/dose	and workers^a^	(118/166)	0–1–6 months	C	141	and mild fever.
Gizaris1993[19]	RV	Healthy adults	17–22 y	0–1–2–12 months	T	100	local pain, headache,
	20 ug/dose		(100/100)	0–1–6 months	C	100	mild fever
Winter1994[18]	RV	Healthy adults	NR (35/80)	0–1–2–12 months	T	59	Unclear
	20 ug/dose			0–1–6 months	C	56	
Marsano1996[13]	RV	Healthy adults^a^	19–62 y	0–1–2 months	T	113	Unclear
	20 ug/dose		(83/147)	0–1–6 months	C	117	
Asli2011[4]	RV	Male Prisoners^a^	Mean age 34 y	0–7–28–56 days	T	85	Unclear
	20 ug/dose		(169/0)	0–1–6 months	C	84	

T = accelerated schedule, C = standard schedule.

^a^All HBsAg, HBsAb, and HBcAb tests were negative. RV = recombinant vaccine; NR = not reported.

**Table 2:** Overview of hepatitis B vaccine uptake according to vaccination schedule in different at-risk populations.

Ref.	Group	Sample	HBsAb positive rate after the initial dose					anti-HB antibody levels after initial dose (1:) (95%CI or median)				
		size	1^st^	3^rd^	7^th^	12^th^	others	1^st^	3^rd^	7^th^	12^th^	others
Yuan2004[31]	T	50	32/50	38/50	46/50	44/50	NR	108.6	104.3	56.2	68.3	NR
	C	50	13/50	37/50	47/50	45/49	NR	59.4	74.5	107.6	84.2	NR
Chen2006[29]	T	100	63/99	NR	87/97	NR	70/96^a^	97(69–125)	NR	107(77–137)	NR	37(26–47) ^a^
	C	100	27/100	NR	91/98	NR	75/95^a^	15(11–19)	NR	213(152–273)	NR	89(63–114) ^a^
Yuan2006[30]	T	150	91/148	NR	NR	113/146	86/139^b^	63(53–72)	NR	NR	74(62–85)	25(21–29) ^b^
	C	150	34/149	NR	NR	117/145	94/141^b^	12(10–14)	NR	NR	115(98–132)	67(56–77) ^b^
Wahl1988[26]	T	27	13/27	23/27	27/27	NR	NR	NR	NR	83	NR	NR
	C	26	1/26	11/26	25/25	NR	NR	NR	NR	430	NR	NR
Ricciardi1990[21]	T	50	NR	NR	42/50	NR	NR	NR	NR	383	NR	NR
	C	65	NR	NR	63/65	NR	NR	NR	NR	704	NR	NR
Hess1992[22]	T	143	44/138	102/125	112/125	103/121	NR	11.6	160	173	5608	NR
	C	141	25/137	86/123	108/113	111/119	NR	8.3	40.0	2877	442	NR
Gizaris1993[19]	T	100	34/94	NR	NR	93/94	NR	2.2	NR	NR	16269.7	NR
	C	100	33/98	NR	NR	97/98	NR	2.1	NR	NR	1188.0	NR
Winter1994[18]	T	59	NR	47/54	47/53	52/54	NR	NR	NR	NR	NR	NR
	C	56	NR	39/56	52/55	42/50	NR	NR	NR	NR	NR	NR
Marsano1996[13]	T	113	70/112	101/105	95/98	NR	NR	NR	132.7	346.7	NR	NR
	C	117	60/114	82/112	106/107	NR	NR	NR	23.9	4263.8	NR	NR
Asli2011[4]	T	85	19/85	NR	67/85	NR	NR	21.6(63)	NR	141.24(110.15)	NR	NR
	C	84	4/84	NR	71/76	NR	NR	5.08(29.8)	NR	194.3(91.73)	NR	NR

T = accelerated schedule, C = standard schedule.

^a^after 22 months

^b^after 36 months.

CI = confidence interval; NR = not reported.

### Quality Assessment

Among included studies (S1 and S2 Figs), four applied a random table [4, 22, 26, 31], but the remainder did not report any details of random-sequence generation. Concealment of allocation was an undefined risk in the included studies because it was not reported. Six studies had low attrition bias [4, 13, 19, 21–22, 26], and the others were unclear. Reporting, performance, and detection biases were low.

### Comparison of seroprotection rates

**Fig 2:**
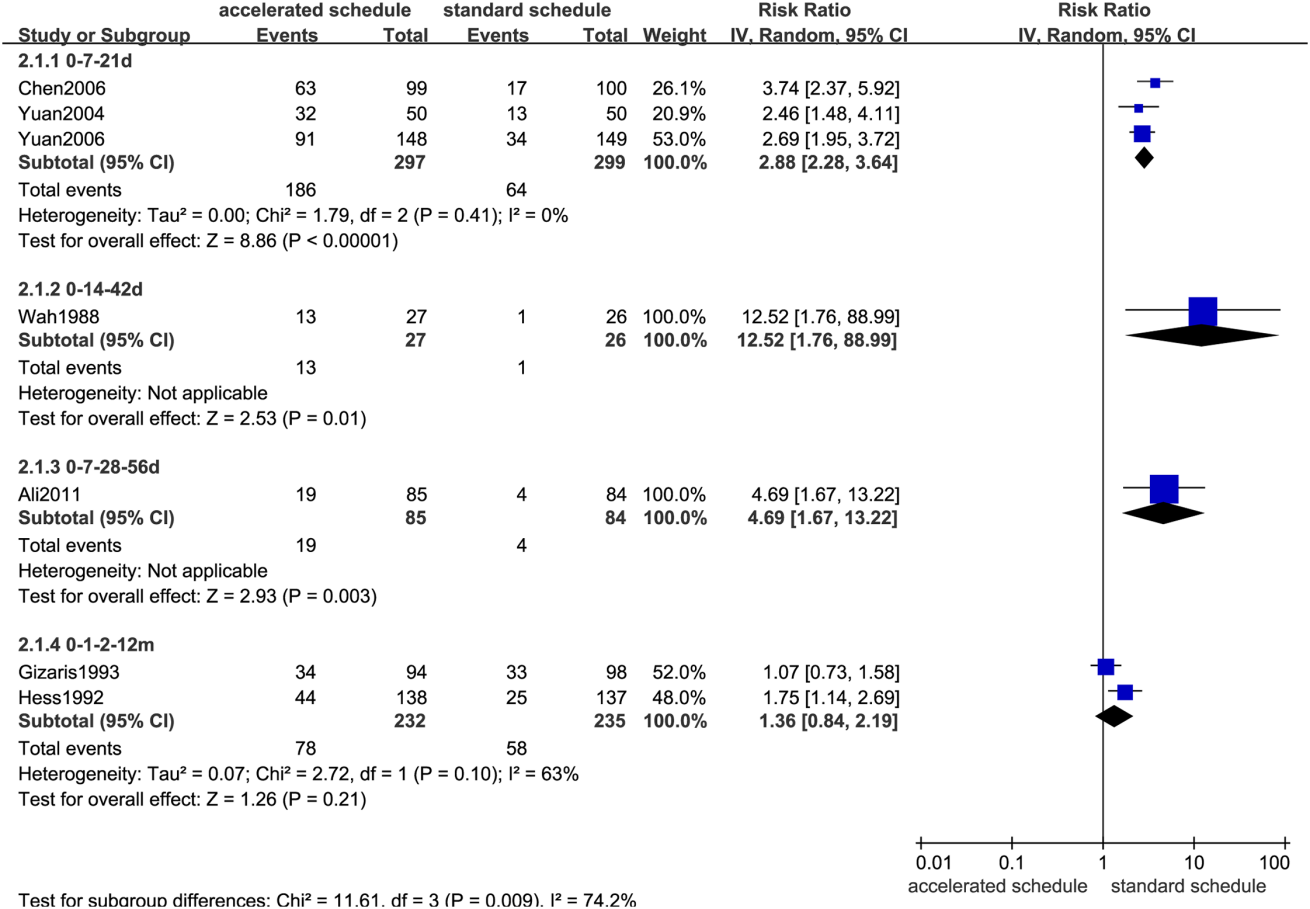
Forest plots showing protective rate comparisons between accelerated and standard schedules for intention-to-treat analysis at 1 month after initial dose.

**Fig 3:**
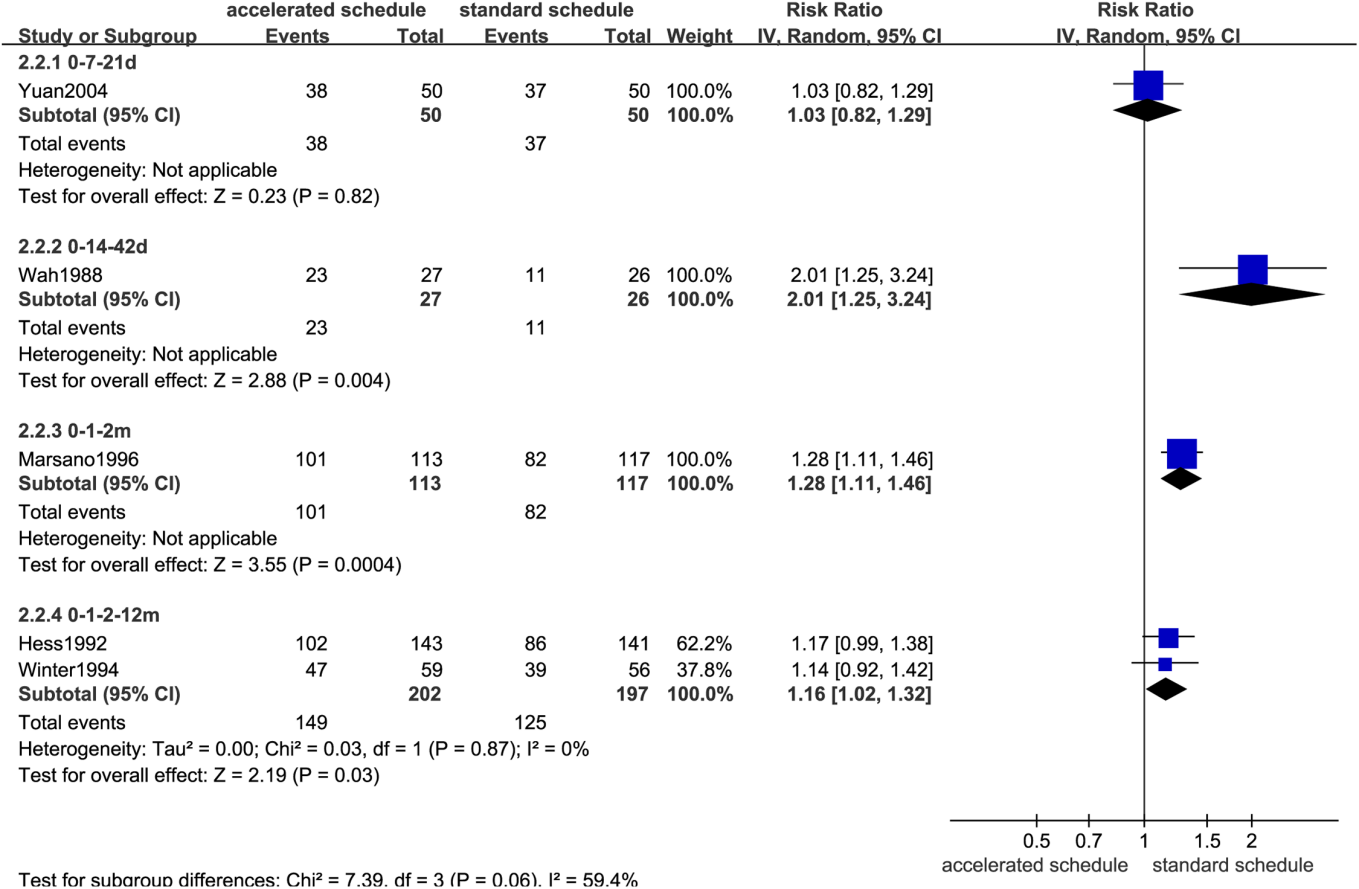
Forest plots showing protective rate comparisons between accelerated and standard schedules for intention-to-treat analysis at 3 month after initial dose.

**Fig 4:**
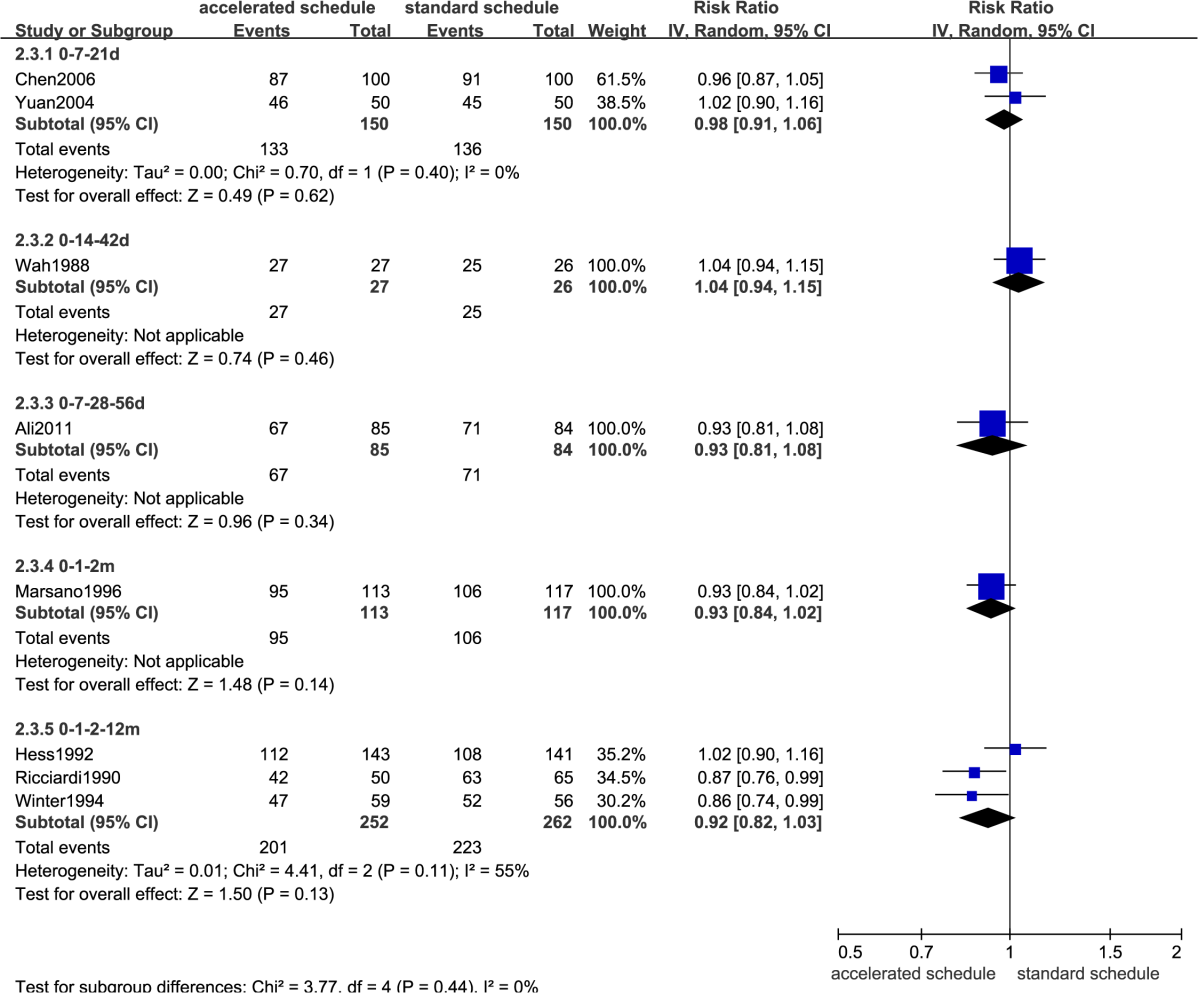
Forest plots showing protective rate comparisons between accelerated and standard schedules for intention-to-treat analysis at 7 month after initial dose.

**Fig 5:**
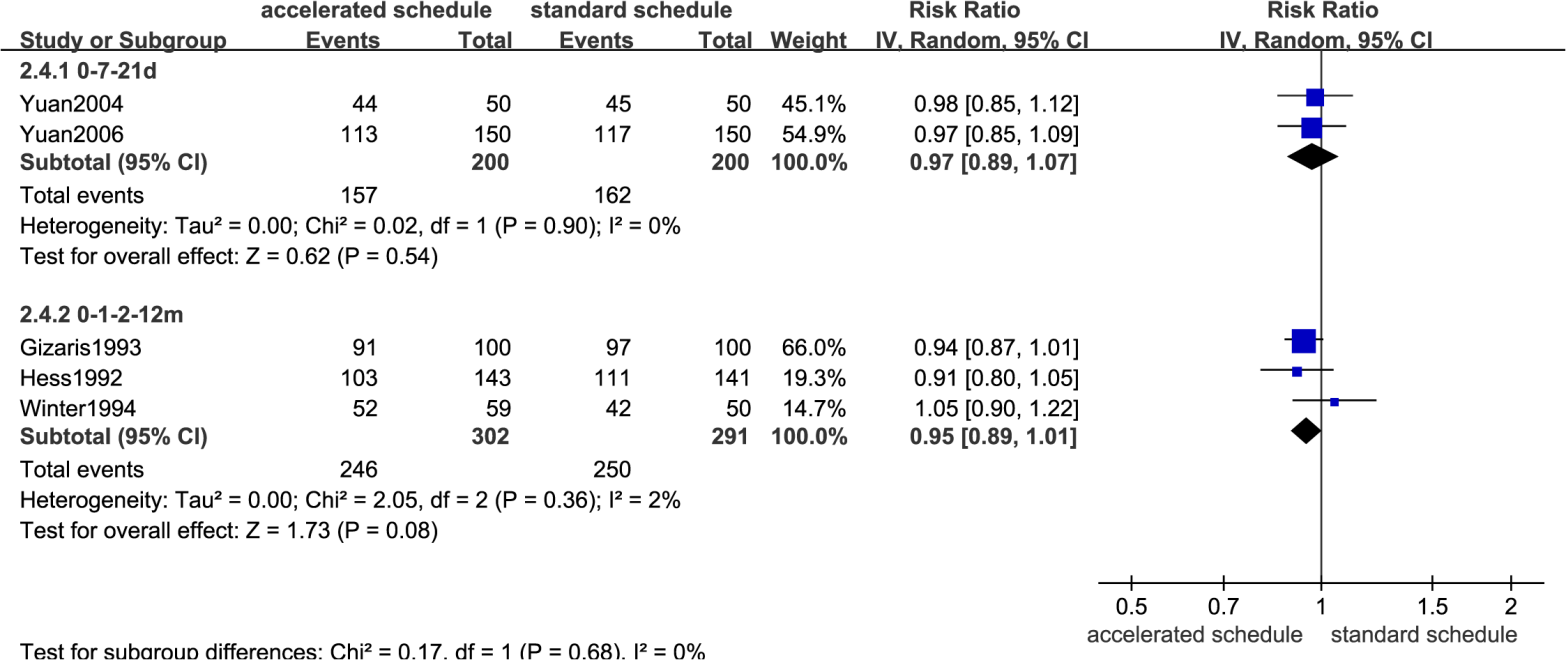
Forest plots showing protective rate comparisons between accelerated and standard schedules for intention-to-treat analysis at 12 month after initial dose.

**Fig 6:**
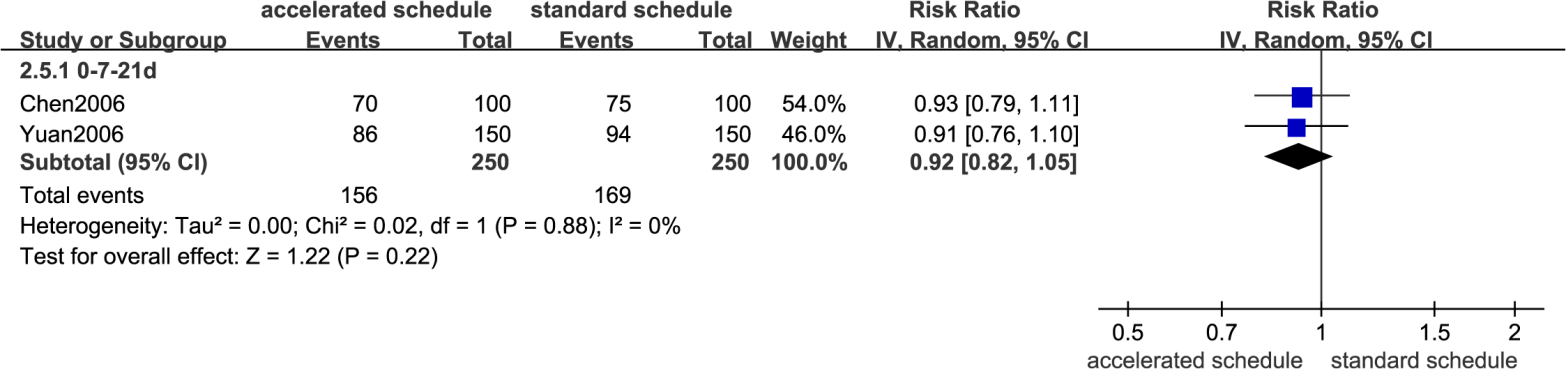
Forest plots showing protective rate comparisons between accelerated and standard schedules for intention-to-treat analysis at 22 month after initial dose.

**Fig 7:**
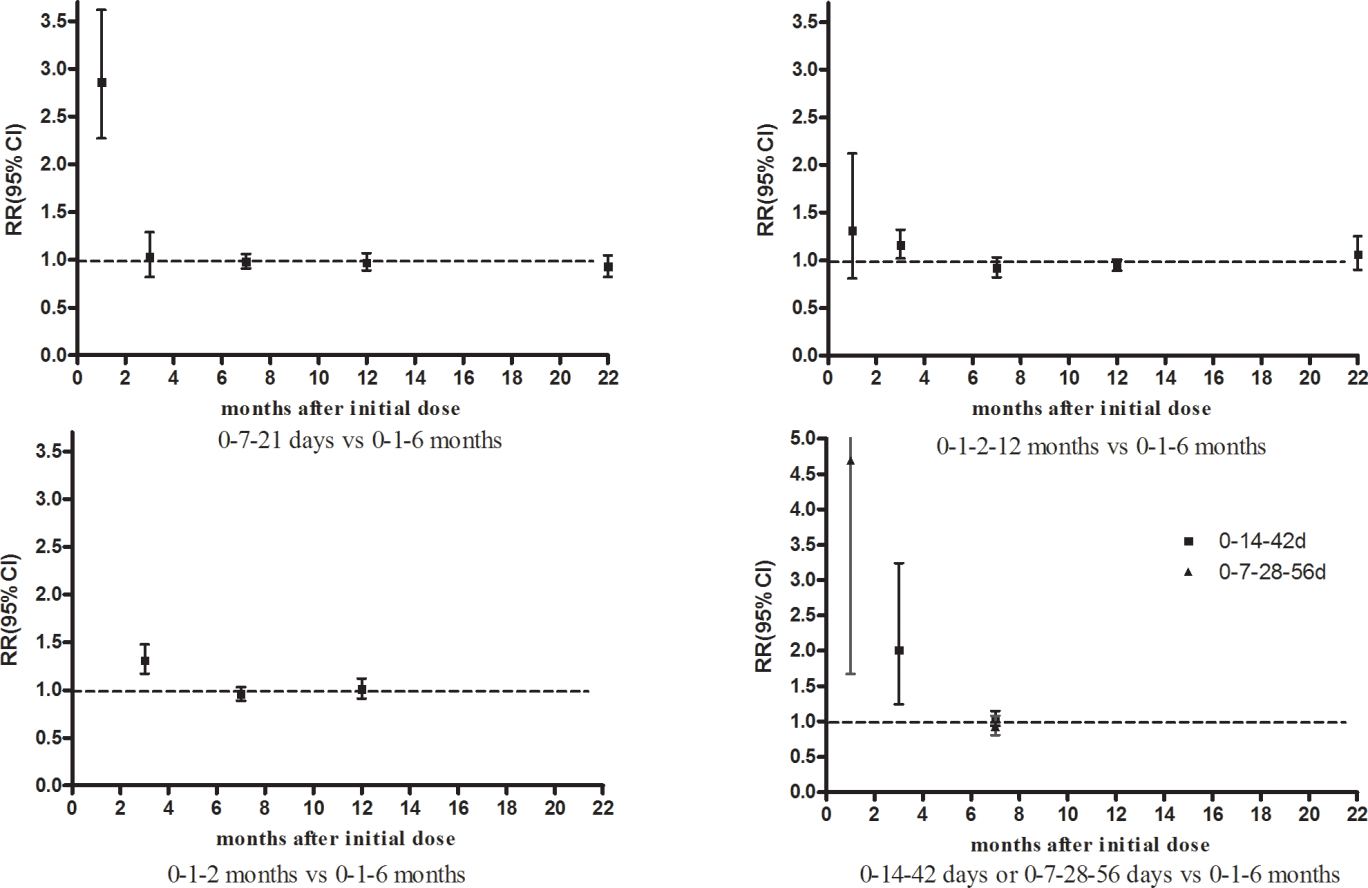
Seroprotection rate changes for different vaccination schedules according to months after initial dose.

Dose timing and protective response to vaccine differed between subjects vaccinated according to accelerated (accelerated group) and standard schedules (standard group) (Figs 2–7, S3–S7 Figs, and Table 3). Due to the heterogeneity of many types of accelerated schedules, each type of accelerated group was independently analyzed in meta-analysis to evaluate meta-RR. Generally, higher seroprotection rates were detected in the accelerated group compared with the standard group at the first or third month after the initial dose, including accelerated schedules of 0–7–21 days, 0–7–28–56 days, 0–14–42 days, 0–1–2 months, and 0–1–2–12 months (Table 3), according to ITT analysis or PP analysis.

**Table 3:** Comparison of protective rates according to vaccination schedule in different at-risk populations.

Accelerated	Ref.	Sample	RR (95%CI) (IV, Random)	
schedule		size (ITT/PP)	ITT	PP
1^st^ month after initial dose				
0–7–21 days	3	600/596	2.86(2.27,3.62)	2.88(2.28,3.64)
0–14–42 days	1	53/53	12.52(1.76,88.99)	12.52(1.76,88.99)
0–7–28–56 days	1	169/169	4.69(1.67,13.22)	4.69(1.67,13.22)
0–1–2–12 months	2	480/467	1.31(0.81,2.12)	1.36(0.84,2.19)
3^rd^ month after initial dose				
0–7–21 days	1	100/100	1.03(0.82,1.29)	1.03(0.82,1.29)
0–14–42 days	1	53/53	**2.01(1.25,3.24)**	**2.01(1.25,3.24)**
0–1–2 months	1	230/217	**1.28(1.11,1.46)**	**1.31(1.17,1.48)**
0–1–2–12 months	2	399/358	**1.16(1.02,1.32)**	**1.19(1.06,1.34)**
7^th^ month after initial dose				
0–7–21 days	2	300/295	0.98(0.91,1.06)	0.98(0.92,1.06)
0–14–42 days	1	53/52	1.04(0.94,1.15)	1.00(0.93,1.08)
0–7–28–56 days	1	169/161	0.93(0.81,1.08)	**0.84(0.74,0.96)**
0–1–2 months	1	230/205	0.93(0.84,1.02)	0.98(0.94,1.02)
0–1–2–12 months	3	514/461	0.92(0.82,1.03)	**0.92(0.87,0.98)**
12^th^ month after initial dose				
0–7–21 days	2	400/390	0.97(0.89,1.07)	0.96(0.88,1.05)
0–1–2–12 months	3	593/525	0.95(0.89,1.01)^a^	1.02(0.97,1.07) ^a^
>22 months after initial dose				
0–7–21 days	2	500/471	0.92(0.82,1.05)	0.93(0.82,1.04)

^a^13 month for the trial group. RR = relative risk; CI = confidence interval; IV = inverse variance; ITT = intention-to-treat; PP = per-protocol. Bold fonts indicate statistical significance (*P*<0.05).

However, there were no statistically significant differences in seroprotection rates between the accelerated and standard groups at ≥7 months after the initial dose, except that PP analysis (S5 and S7 Figs) showed that the 0–7–28–56 day (RR = 0.84, 95%CI: 0.74–0.96) and 0–1–2–12 month (RR = 0.92, 95%CI: 0.87–0.98) accelerated schedules had lower seroprotection rates than the standard group at 7 months after the initial dose.

### Comparison of anti-HBs levels

**Fig 8:**
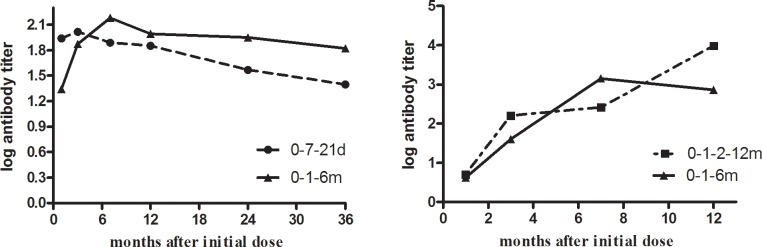
Log_10_ antibody titer changes for different vaccination schedules according to months after initial dose.

Forest plots comparing anti-HB levels are not shown because very few studies could be included in the analysis. Table 4 and Fig 8 show changes in anti-HB levels in accelerated and standard groups at different time intervals after the initial dose. Anti-HB levels in the accelerated group with 0–7–21 day and 0–7–28–56 day schedules were higher than the standard group 1 month after the initial vaccine dose. However, at 7, 12, 24, and 36 months after the initial dose, anti-HB levels in the 0–7–21 day accelerated group were statistically lower than the standard group (Table 4).

**Table 4:** Comparison of anti-hepatitis B antibody levels according to vaccination schedule in different at-risk populations.

Months after initial	Accelerated	Ref	Sample	Mean log10 (95%CI) difference^b^	
dose	schedule		size	M-H, Fixed model	IV, Random model
1^st^ month	0–7–21 days	2	397	**1.71(1.54,1.89)**	**1.71(1.54,1.89)**
	0–7–28–56 days	1	169	**1.44(0.30,2.58)**	**1.44(0.30,2.58)**
7^th^ month	0–7–21 days	1	195	**-0.69(-1.05,-0.34)**	**-0.69(-1.05,-0.34)**
	0–7–28–56 days^a^	1	161	-0.31(-1.71,1.07)	-0.31(-1.71,1.07)
12^th^ month	0–7–21 days	1	291	**-0.44(-0.64,-0.24)**	**-0.44(-0.64,-0.24)**
24^th^ month	0–7–21 days	1	191	**-0.88(-1.23,-0.53)**	**-0.88(-1.23,-0.53)**
36^th^ month	0–7–21 days	1	280	**-0.98(-1.10,-0.86)**	**-0.98(-1.10,-0.86)**

^a^8 months for the trial group.

^b^ per-protocol (PP) analysis. CI = confidence interval; MH = Mantel–Haenszel; IV = inverse variance. Bold fonts indicate statistical significance (*P*<0.05).

Fig 8 summarizes anti-HB levels from all included studies and shows that the standard group had higher log antibody titers than the accelerated 0–7–21 day schedule from the 7^th^ to the 36^th^ month after the initial dose. The accelerated 0–1–2–12 month schedule group had higher anti-HB levels than the standard group at 12 months after the initial vaccine dose.

### Compliance comparison

Only two studies considered compliance rate as an endpoint [4, 22]. Asli et al. [4] found a significantly higher rate of compliance (100%) among male prisoners in the 0–7–28–56 day accelerated group compared to the standard group (90.5%) after a full course of vaccination. However, Hess et al. [22] showed a significantly lower compliance rate (90%) in the 0–1–2–12 month accelerated group than the standard group (99%); notably, the standard group compliance rate was remained higher than the accelerated group even at the third scheduled vaccination (91%).

Meta-analysis of the two studies demonstrated similar compliance rates between accelerated and standard groups when compliance of the 12^th^ month vaccination was not considered (RR = 1.00, 95%CI: 0.84–1.21).

### Sensitivity, subgroup, and funnel plot analyses

Due to the limited number of trials and the low methodological quality in each comparison group, we were unable to perform sensitivity and subgroup analyses or generate funnel plot as anticipated.

### Safety analysis

The most common symptoms, such as mild fever, local pain, diarrhea, and other discomforts, were reported in the included studies, but serious adverse events were not described.

## Discussion

This study used meta-analysis to investigate the beneficial and harmful effects of different hepatitis B vaccination schedules in adults. The main findings of our study are discussed below.

The accelerated schedule is generally appealing because it may increase participant compliance and provide earlier protection for people at high risk of hepatitis B infection [35]. However, it has not been widely used due to concerns that anti-HB seroconversion rates and protection duration may be inferior to the standard schedule [21, 35]. Similarly, our meta-analysis revealed that most accelerated schedules had higher seroprotection rates than the standard schedule the first month, including 0–7–21 day, 0–7–28–56 day, 0–14–42 day, 0–1–2 month, and 0–1–2–12 month schedules. However, there were no statistically significant seroprotection rate differences between individual accelerated schedules and the standard schedule after 6 months, except for PP analysis of 0–7–28–56 day and 0–1–2–12 month schedules (Table 3). These findings were similar to other study results [4, 7], suggesting that longer efficacy follow-ups of accelerated 0–7–21 day [29, 30] or 0–1–2 month [7] schedules could provide additional evidence for seroprotection rates similar to the standard schedule.

Our meta-analysis showed that mean HBsAg antibody titers were significantly higher in the standard group than in the accelerated group after the 6^th^ month, nevertheless GMT values for both schedules were all well above the minimal protection threshold. Moreover, anti-HB titers in accelerated group increased and reached seroprotective levels more rapidly than the standard group [18, 19, 21, 22]. Although fourth booster dose for the 0–1–2 month schedule (Fig 4) has been recommended to decelerate rapidly declining antibody levels [18, 19, 21, 22], completing the schedule with an additional booster is more difficult to ensure the compliance of hard-to-reach populations than completing a standard 0–1–6 month schedule [22].

The effectiveness and suitability of vaccination protocols should not be based entirely on seroprotection rates. Individual compliance to receive the full vaccine course should also be taken into account when evaluating protocol efficacy. In this analysis, only two studies considered compliance rates as endpoints [4, 22]. Meta-analysis of these studies demonstrated that, when compliance rates in the 12^th^ month were not considered, the accelerated and standard groups had similar compliance rates (RR = 1.00, 95%CI: 0.84–1.21). In fact, the two studies had diametrically opposed conclusions. Asli et al. [4] found a significantly higher rate of compliance (100%) in the accelerated group schedule of 0–7–28–56 days compared to the standard schedule (90.5%) among male prison inmates who completed the full course of vaccination, while Hess et al. [22] showed that medical students and health-care workers had a significantly lower compliance rate (91%) in the accelerated group (0–1–2 months) compared to the standard group (99%). One possible explanation is that the study by Asli, et al. included male prisoners as subjects, while the study by Hess, et al. recruited healthy people [27, 28]. This finding suggests that higher compliance rates were closely related to short-term centralized management of risk groups rather than accelerated vaccination schedules.

This meta-analysis had several limitations, most significantly the number of included studies and the variety of shortened schedules. These limitations could have impacted heterogeneity and sensitivity analyses and restricted our interpretation of the results as evidence for future practice. Therefore, we were not able to perform sensitivity/subgroup and funnel plot analyses as planned. Some factors, such as male dominance (57.03%, 1026/1799) limit the generalizability of the results. Second, long-term effects were difficult to obtain using RCTs, especially for certain time points. Third, compliance rate calculations were based on the included RCT studies; the numerator depended on the number of study participants lost to follow-up as well as shedding cases and might be different from studies that include follow-ups of the natural population. Finally, insufficient information would bias results such as allocation concealment [10, 20], lab results [23], and compliance at some time points.

## Conclusions

The standard vaccination program appears to be more efficient in terms of sustained antibody levels compared to accelerated schedules without booster doses. Rapid seroconversion and immediate protection in the short term can make it possible for high-risk groups to use accelerated schedules, but the long-term protection and effectiveness of the primary accelerated schedule doses should be recognized in the future.

## Supporting Information

S1 Data PRISMA 2009 Checklist. (DOC)

S2 Data List of excluded studies. (DOC)

S1 Fig Risk of bias among included studies. (TIF)

S2 Fig Summary of bias risk among included studies. (TIF)

S3 Fig Forest plots comparing protective rates of accelerated and standard schedules for pre-protocol analysis at 1 month after initial dose. (TIF)

S4 Fig Forest plots comparing protective rates of accelerated and standard schedules for pre-protocol analysis at 3 month after initial dose. (TIF)

S5 Fig Forest plots comparing protective rates of accelerated and standard schedules for pre-protocol analysis at 7 month after initial dose. (TIF)

S6 Fig Forest plots comparing protective rates of accelerated and standard schedules for pre-protocol analysis at 12 month after initial dose. (TIF)

S7 Fig Forest plots comparing protective rates of accelerated and standard schedules for pre-protocol analysis at 22 month after initial dose. (TIF)
