# Supplementary material for: Comparison of Accelerated and Standard Hepatitis B Vaccination Schedules in High-Risk Healthy Adults: A Meta-Analysis of Randomized Controlled Trials
Source: PLoS One. 2015 Jul 21;10(7):e0133464. doi: 10.1371/journal.pone.0133464 (PMC4510064; doi:10.1371/journal.pone.0133464)
Supplement: S2 Data — (DOC) [file pone.0133464.s002.doc]

**Supplemental Data S2.** List of excluded studies

**Chinese articles**

- *Studies including children only or not allowing to separate children from adults:*

Deng Y *et al*. Guangdo Med J 1991;1:23-24

Liu L. Clin Focus 1995;16:742

Chen Z *et al*. J North Chin Coal Med Uni 2000;6:634

Yuan JM et al. Nurs J Chin People Liber Army 2004;10:67-68

Chen SZ *et al*. World Chin J Digest 2006;19:1884-88

Chen SZ *et al*. World Chin J Digest 2007;16:1865-67

Deng S JM et al. Pract Prevent Med 2011;3:446-48

- *Studies not allocating randomly or uncertain:*

Zhang QY *et al.* Chin J Virol 1991;S1:108-113

Chen YG *et al.* Guangxi J Prevent Med 1996;2:106

Yuan JM *et al.* Shangdong Med 1998;5:63

Yuan JM *et al.* Heilongjiang J Nursing 1998;11:78

Li JL*.* Guangxi Med 1999;5:37-40

Yu YQ *et al.* New Med 2001;3:159-60

Ren P *et al*. Chin J School Doctor 2001;2:107

Ren P *et al.* Chin J School Health 2002;2:129

- *Studies without concurrent comparisons:*

Mu RC *et al.* Chongqing Med 1991;6:336

Zhao LS *et al*. J Pract Med 1992;3:15-16

LU YZ*.* Henan J Prevent Med 2004;2:89-91

Chen YZ *et al*. Chin J Vaccine and Immunization 2005;11(2):100-5

Yu SF *et al*. Dis Surveillance 2012;8:604-6

Xu F *et al*. Dis Surveillance 2013;1:38-41

Chen SY *et al*. Chin J Prevent Med 2013;2:96-98

- *Studies without standard schedule as a control:*

Song JJ *et al.* Shanghai J Immun 1991;4:247-48

Pang ZZ *et al*. Chin J Vaccine and Immunization 2006;12(2):109-11

- *Studies without complete information:*

Kong Y*.* Herald Med 2001;2:110

Cheng P *et al.* Pract J Hepatol 2003;4:196

- *Studies translating from English to chinese:*

Fang XH*.* Guangdong J Health Epidemic Prevent 1990;4:39-41

Li XX*.* Foreign Med Sci (Section of Bilogics for Pro phylaxis, Diagn and Therapy) 1996;3:129

**English articles**

- *Studies including children only or not allowing to separate children from adults:*

Giammanco G *et al*. Postgrad Med J 1987;63(s2):151-54

Greenberg DP. Pediatr Infect Dis J 1994;13(4):339-40

Kallinowski B *et al*. Transplant Proc 1998;30(3):797-99

Chowdhury A et al. World J Gastroenterol 2005;11(7):1037-39

- *Studies not allocating randomly or uncertain:*

Bommer J *et al.* Dtsch Med Wochenschr 1983 ;108(48) :1823-26

Carreno V *et al.* Clin Nephrol 1985;24(5):215-20

Bruguera M *et al.* Postgrad Med J 1987;63(S2):155-58

Jilg W *et al.* J Infect Dis 1989;160(5):766-69

Bruguera M *et al.* Vaccine 1990;S8:S47-S49

Harries AD *et al.* J Infect 1991;23(3):251-54

EI-Reshaid K *et al*. Vaccine 1994;12(3):223-34

Christensen PB *et al.* Vaccine 2004;22(29-30):3897-901

Hwang LY *et al*. J Infect Dis 2010;202(10):1500-09

Tran TQ *et al.* Vaccine 2012;30(2):342-49

- *Studies without concurrent comparisons:*

Marchou B *et al.* J Infect Dis 1995;172(1):258-60

Asboe D *et al*. Genitourin Med 1996;72(3):210-12

Wright NM *et al.* Commun Dis Public Health 2002;5(4):324-26

McMillan A*.* Int J STD AIDS 2005;16(9):633-35

Ghadiri K *et al*. J Res Med Sci 2012;17(10):934-37

- *Studies without standard schedule as a control:*

Scheiermann N *et al.* Vaccine 1990;S8:S44-S46

Bryan JP, et al. J Infect Dis 1991; 163(6):1384-5.

Rustgi VK *et al*. Vaccine 1995;13(17):1665-68

Saltoglu N *et al*. Ann Clin Microbiol Antimicrob 2003;2:10

Tarhan MO *et al*. Med Sci Monit 2006;12(11):R467-70

Hernandez-Bernal F *et al*. Hum Vaccin 2011;7(10):1026-36

De Vries-Sluijs TE *et al.* J Infect Dis 2011;203(7):984-91

- *Reply letters no studies:*

Jaqueti J *et al.* Med Clin (Barc) 1990;94(14):559

Serra DM *et al*. Med Clin (Barc) 1990;94(16):637-38

Bruguera M *et al*. Med Clin(Barc) 1990;94(19):757

Agladioglu SY *et al.* Infection 2011;39(5):489-90

- *Studies about hepatitis A vaccine:*

Westblom TU *et al.* J Infect Dis 1994;169(5):996-1001

- *Studies in non-RCT:*

Hussain Z *et al.* World J Gastroenterol 2005;11(45):7165-68

Sheffield JS *et al.* Obstet Gynecol 2011;117(5):1130-35

- *Studies without complete information:*

Gutierrez GJ *et al.* Rev Clin Esp 1986;178(2):66-69

Wouters K *et al.* Vaccine 2007;25(10):1893-1900

Nyamathi AM *et al.* J Viral Hepat 2009;16(9):666-73
