# Supplementary figures and images for: Comparison of Accelerated and Standard Hepatitis B Vaccination Schedules in High-Risk Healthy Adults: A Meta-Analysis of Randomized Controlled Trials
Source: PLoS One. 2015 Jul 21;10(7):e0133464. doi: 10.1371/journal.pone.0133464 (PMC4510064; doi:10.1371/journal.pone.0133464)

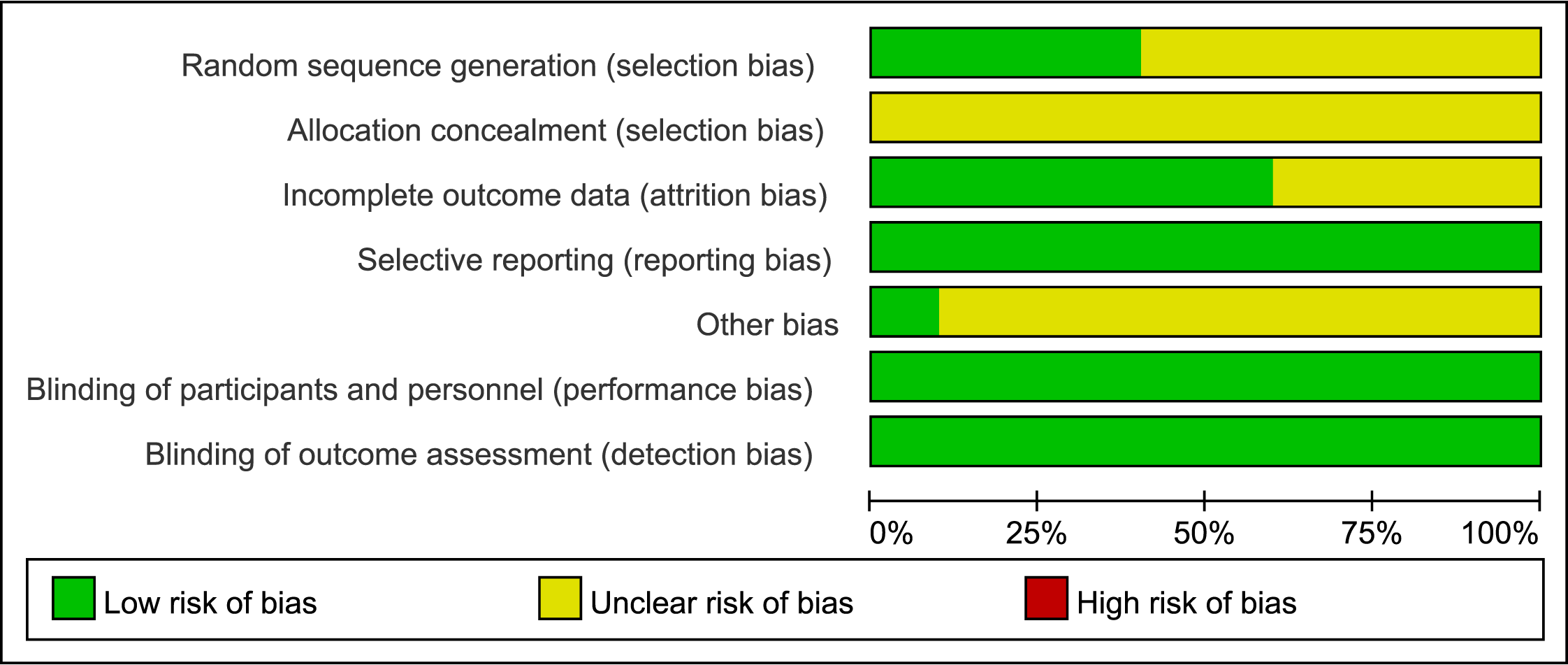

Supplement: S1 Fig — (TIF) [file pone.0133464.s003.tif]

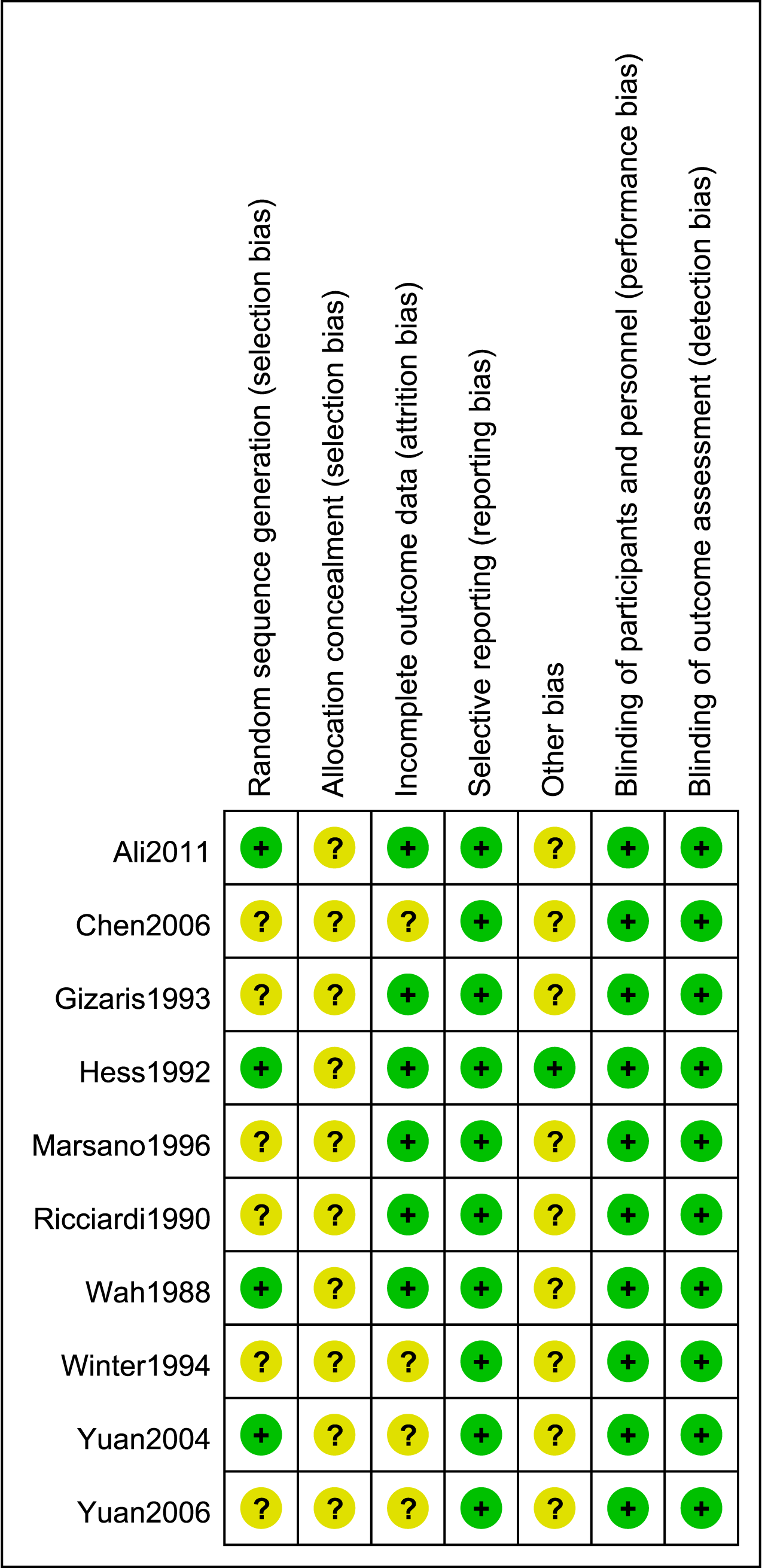

Supplement: S2 Fig — (TIF) [file pone.0133464.s004.tif]

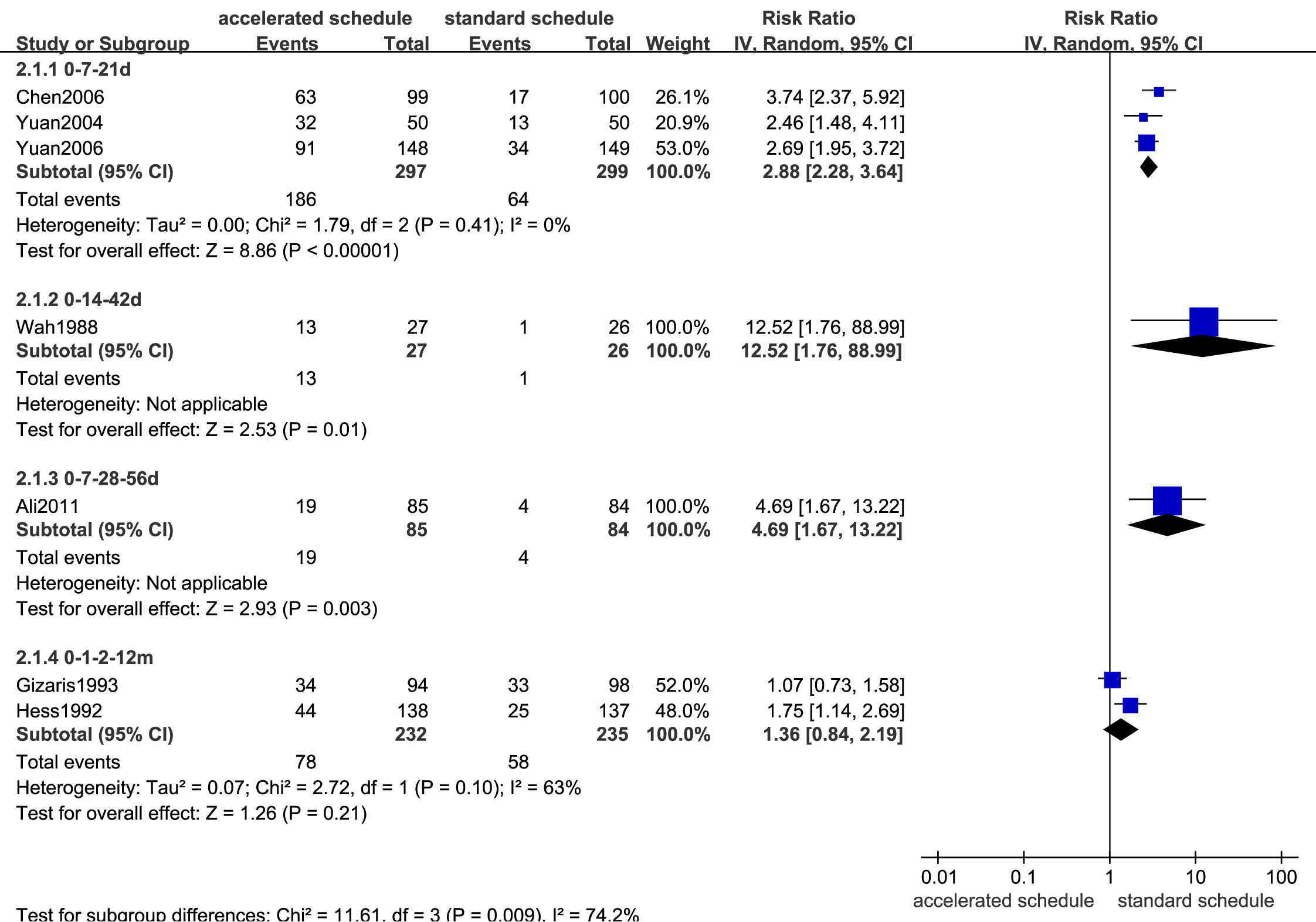

Supplement: S3 Fig — (TIF) [file pone.0133464.s005.tif]

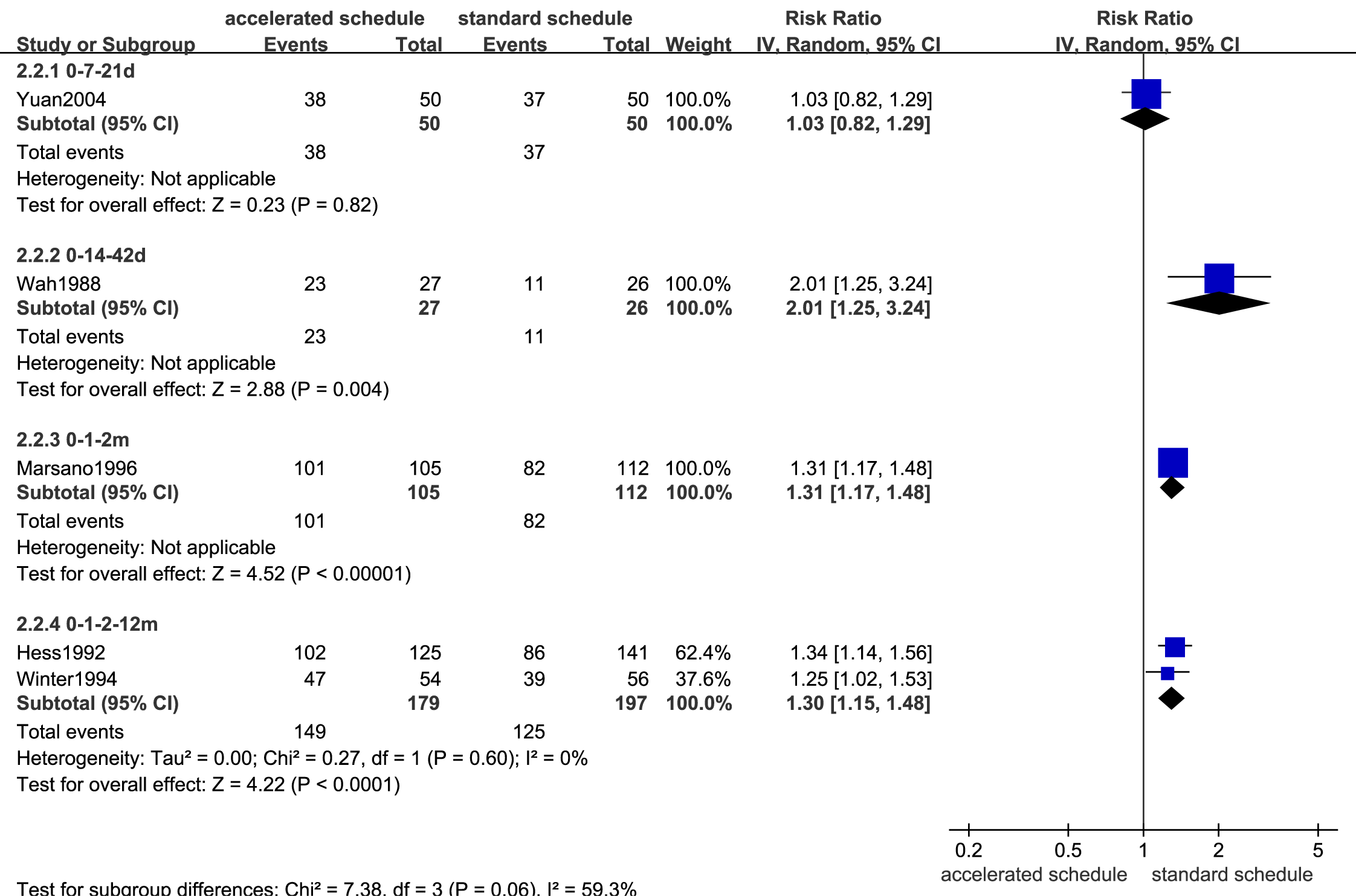

Supplement: S4 Fig — (TIF) [file pone.0133464.s006.tif]

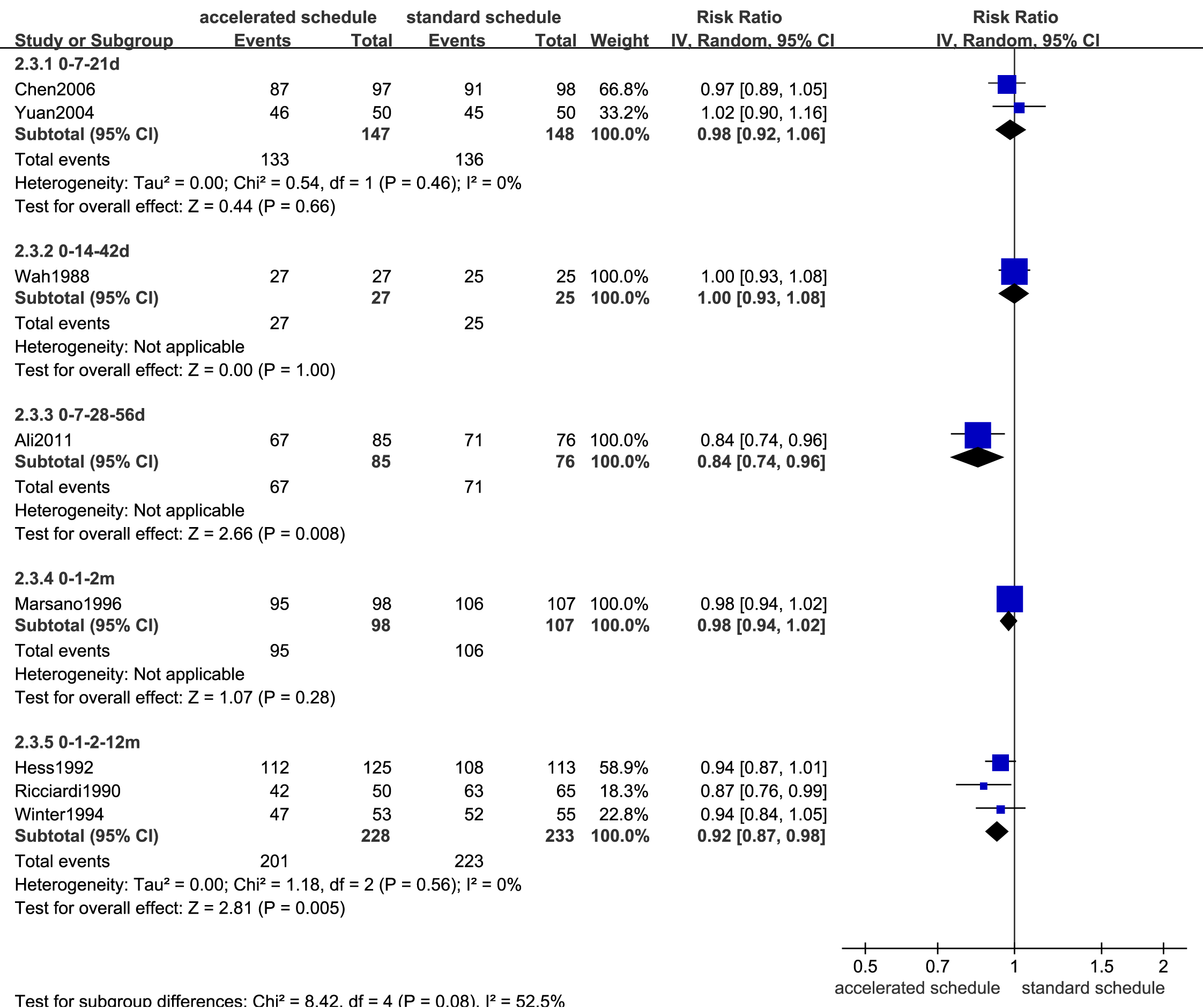

Supplement: S5 Fig — (TIF) [file pone.0133464.s007.tif]

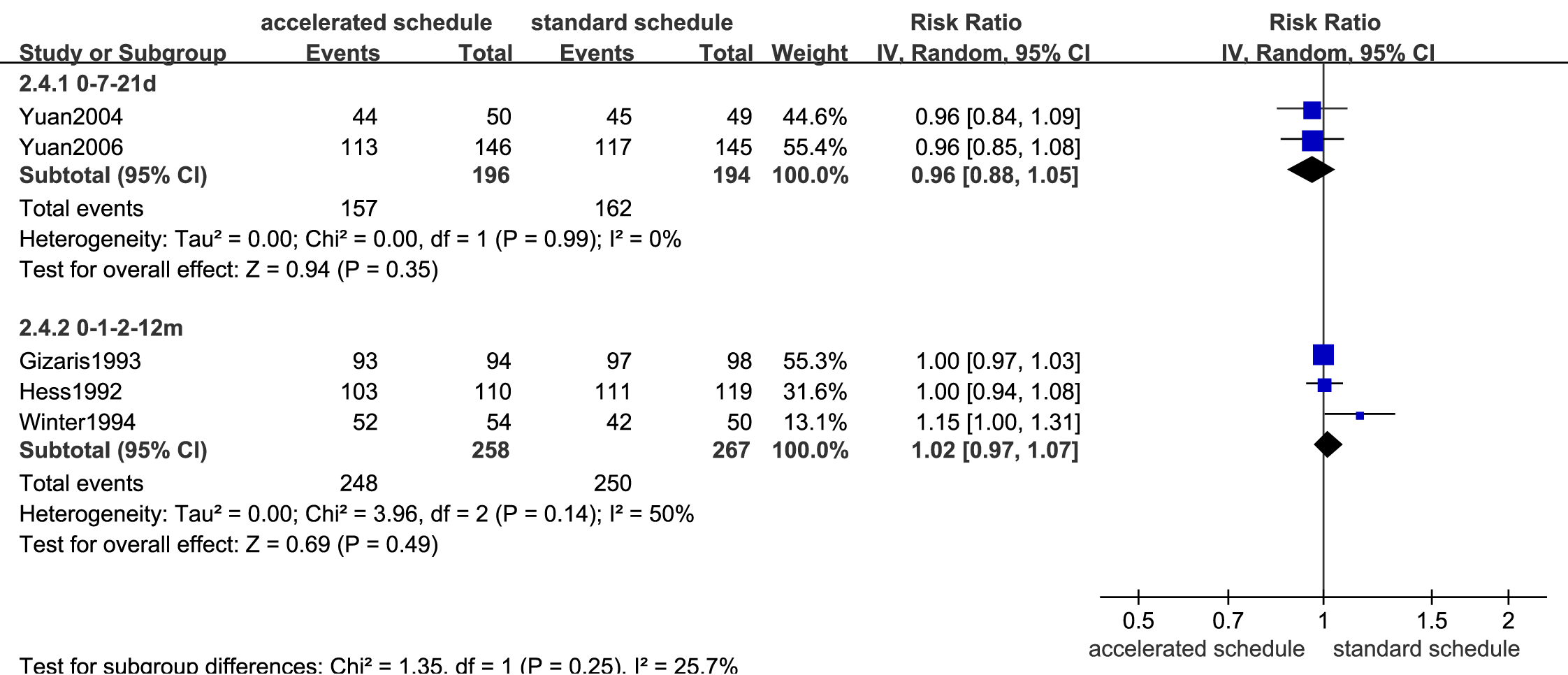

Supplement: S6 Fig — (TIF) [file pone.0133464.s008.tif]

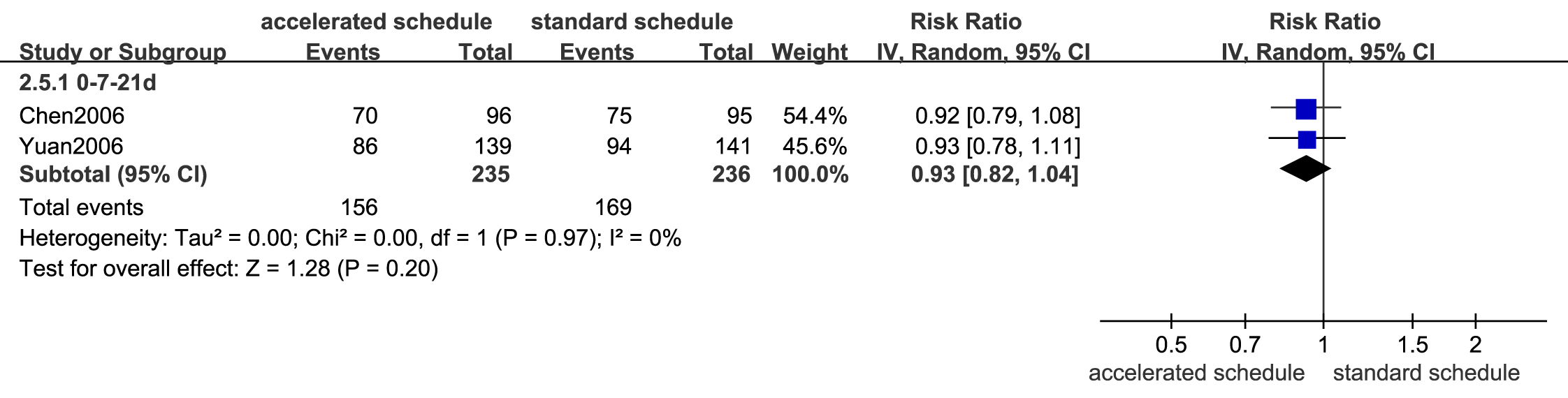

Supplement: S7 Fig — (TIF) [file pone.0133464.s009.tif]
